# Quantitative RT-PCR assay for high-throughput screening (HTS) of drugs against the growth of *Cryptosporidium parvum in vitro*

**DOI:** 10.3389/fmicb.2015.00991

**Published:** 2015-09-22

**Authors:** Haili Zhang, Guan Zhu

**Affiliations:** Department of Veterinary Pathobiology, College of Veterinary Medicine & Biomedical Sciences, Texas A&M UniversityCollege Station, TX, USA

**Keywords:** *Cryptosporidium parvum*, *in vitro*, qRT-PCR, high-throughput screening, assay development, paromomycin

## Abstract

Our laboratory has previously developed a qRT-PCR assay to assess drug efficacy on the growth of *Cryptosporidium parvum in vitro* by detecting the levels of parasite 18S rRNA. This approach displayed up to four orders of magnitude of linear dynamic range and was much less labor-intensive than the traditional microscopic methods. However, conventional qRT-PCR protocol is not very amendable to high-throughput analysis when total RNA needs to be purified by lengthy, multi-step procedures. Recently, several commercial reagents are available for preparing cell lysates that could be directly used in downstream qRT-PCR analysis (e.g., Ambion Cell-to-cDNA kit and Bio-Rad iScript sample preparation reagent). Using these reagents, we are able to adapt the qRT-PCR assay into high-throughput screening of drugs *in vitro* (i.e., 96-well and 384-well formats for the cultivation of parasites and qRT-PCR detection, respectively). This qRT-PCR protocol is able to give a >150-fold linear dynamic range using samples isolated from cells infected with various numbers of parasites. The new assay is also validated by the NIH-recommended intra-plate, inter-plate, and inter-day uniformity tests. The robustness and effectiveness of the assay are also confirmed by evaluating the anti-cryptosporidial efficacy of paromomycin and by a small scale screening of compounds.

## Introduction

*Cryptosporidium* is a genus of protozoan pathogens belonging to the Phylum Apicomplexa that infect humans and/or animals. Among them, *C. parvum* and *C. hominis* are the two major species causing water-borne and food-borne diarrheal illness in humans (Baldursson and Karanis, 2011; Budu-Amoako et al., 2011). These two and some other species, including *C. meleagridis, C. muris, C. canis*, and *C. felis*, also cause opportunistic infections in AIDS patients (AIDS-OIs) that can be chronic and fatal (Thompson et al., 2005; Feasey et al., 2011; O'connor et al., 2011; Shirley et al., 2012). A more recent multi-country study also revealed that *Cryptosporidium* is one of the top five diarrheal-causing pathogens in children in developing countries, in which the infection increased death rate and negatively affected the growth of children (Kotloff et al., 2013). However, options to treat cryptosporidiosis are limited. In fact, only a single drug, nitazoxanide (NTZ) is approved by the U.S. Food and Drug Administration (FDA) to treat human cryptosporidial infection in immunocompetent patients. Moreover, NTZ is ineffective in treating cryptosporidiosis in AIDS patients, and its efficacy in immunocompetent patients is also debatable (Cabada and White, 2010; Feasey et al., 2011; O'connor et al., 2011; Checkley et al., 2015).

Two approaches are commonly employed in early stages of drug discovery against infectious diseases. Hits and leads may be identified from new chemical entities by directly testing their efficacies against an *in vitro* or *in vivo* model of disease (traditional methods), or by screening compounds against drug targets from a pathogen such as essential enzymes or receptors (target-based methods). In the later case, identified hits/leads will still be evaluated on their *in vitro* efficacy before being advanced to *in vivo* drug testing. Therefore, the availability of a good *in vitro* disease model is critical in drug discovery against diseases including cryptosporidiosis. *In vitro* models of cryptosporidial infection are available, including the cultivation of *C. parvum* in HCT-8 or Caco-2 cells (Arrowood, 2002; Cai et al., 2005; Karanis and Aldeyarbi, 2011). However, there is a need for developing new methods that can efficiently evaluate the parasite growth in response to drug treatment in high-throughput or high-volume format. Conventional microscopic examination to count the numbers of parasites stained chemically or by immunofluorescence labeled antibodies is highly labor-intensive and sometimes subjective, making it unsuitable for testing a large number of drugs. ELISA and chemiluminescence immunoassay methods were reported, but they appeared to have relatively narrow linear dynamic ranges of detection (i.e., the ratio between the largest and smallest signal values of detection is proportional to the difference between the two samples, such as the relationship between the OD values and concentrations of an antigen in an ELISA assay) and heavily relying on the quality and availability of specific antibodies.

More recent approaches include quantitating parasite loads by quantitative PCR (qPCR), qRT-PCR and automated fluorescence imaging analysis that are generally more advantageous than the earlier methods. Among them, the qRT-PCR method originally developed in our laboratory evaluates drug efficacy by detecting the relative levels of parasite 18S rRNA transcripts, which displays up to four orders of magnitude of linear dynamic range between the cycle threshold (C_T_) value and the logarithm of the number of oocyst inoculum (Cai et al., 2005; Ctrnáctá et al., 2010; Zhang et al., 2012). However, classic qRT-PCR protocol is not very amendable to the high-throughput or high-volume analysis when total RNA needs to be purified by lengthy, multi-step procedures.

Here we report an improved and simplified qRT-PCR protocol for evaluating of drug efficacies against the growth of *C. parvum in vitro*. This new protocol takes advantage of the commercially available lysis buffers to prepare cell lysates that could be directly used in downstream qRT-PCR analysis. We also re-optimized the primers and procedures, and adapted the *in vitro* cultivation of parasite in 96-well plates and real-time RT-PCR detection in 384-well plates, making the protocol suitable for high-throughput or high-volume evaluation of drug efficacy against the growth of *C. parvum in vitro*.

## Materials and methods

### Cultivation of parasite *in vitro*

Fresh oocysts of *C. parvum* (Iowa strain) were purchased from Bunch Grass Farm (Deary, ID). Oocysts were further purified by a Percoll-based gradient centrifugation method and surface sterilized with 10% bleach for 7 min on ice, followed by washes with phosphate-buffered saline (PBS). Only *C. parvum* oocysts less than 3 months old were used in all experiments. An ileocecal colorectal adenocarcinoma cell line (HCT-8, ATCC # CCL-244) was used to host the growth of *C. parvum in vitro*. One day before the inoculation, HCT-8 cells were seeded in 96-well plates (2.5 × 10^4^ cells/well) containing RPMI 1640 medium supplied with 10% fetal bovine serum (200 μL medium/well in all experiments) and allowed to grow overnight at 37°C under 5% CO_2_ atmosphere until they reached ~90% confluence. The infection and growth of *C. parvum in vitro* in 96-well plates were examined by differential interference contrast (DIC) microscopy and immunofluorescence (IF) labeling using a rabbit polyclonal antiserum raised against *C. parvum* sporozoite total membrane proteins (Zeng and Zhu, 2006; Zeng et al., 2006; Fritzler et al., 2007), and by qRT-PCR (as described below).

For generating standard curves, host cells were inoculated with five-fold serial dilutions of parasite oocysts (i.e., 4 × 10^4^–64 oocysts/well), responding to the ratios between parasite and host cells ranging from ~1:1 to 1:625. For drug testing, host cells were infected with 2 × 10^4^ oocysts per well (i.e., ratio 1:2). After inoculation, parasite oocysts were allowed to undergo excystation and invasion into host cells for 3 h at 37°C. Free parasites and oocyst walls in the medium were removed from the plates by an exchange of the culture medium. Drugs at specified concentrations were added into the culture at this time point (i.e., immediately after the medium exchange). Parasite-infected cells were then incubated at 37°C for additional 41 h (i.e., total 44 h infection time). For dose response of paromomycin (PRM), 3 × serial diluted PRM from 30 to 800 μg/mL (i.e., 42–1120 μM) was added to infected cells after 3 h post-infection (hpi) and the treatment lasted for 41 h. At least two independent experiments were conducted for every experimental condition, each including two replicates drugs and three replicates for negative and positive controls.

### Preparation of cell lysates

Plates containing HCT-8 cells infected with *C. parvum* for 44 h and uninfected cells grown under the same condition were first centrifuged in a Sigma 2–5 plate centrifuge (Sigma Laborzentrifugen GmbH, Germany) for 10 min at 1000 × g to ensure that free merozoites in the medium were firmly settled on the bottom of the wells. Medium was removed by gentle flicking the plate over a container placed in the biosafety cabinet, followed by two gentle washes with PBS. After removal of PBS and blotting plates on paper towels to remove remaining drops, plates were placed on ice in a rectangle bucket. An eight-channel electronic pipette (Rainin Instrument, Oakland, CA) was used to add PBS and lysis buffer in subsequent procedures.

For extracting total RNA used in high-throughput screening (HTS), 200 μL of ice-cold Bio-Rad iScript qRT-PCR sample preparation reagent (lysis buffer) (Bio-Rad Laboratories, Hercules, CA) was added into each well. Plates were sealed with adhesive and heat sealing films and the bucket containing racked plates on ice was secured in a multi-tube vortexer (VX-2500, VWR International, Radnor, PA) and subjected to vortex for 20 min at the speed set at 7. Plates were then incubated at 75°C for 15 min, followed by centrifugation (5 min, 2000 × g) to settle down cell debris. Supernatants were used immediately in subsequent qRT-PCR reactions or the plates were stored at −80°C until use.

### Real-time qRT-PCR assay

The levels of 18S rRNA transcripts from *C. parvum* and host cells (referred to as Cp18S and Hs18S) were detected by real-time qRT-PCR method using qScript™ one-step SYBR green qRT-PCR kit (Quanta Biosciences, Gaithersburg, MD). Cell lysates prepared as described above were diluted by 100 and 2000 folds for detecting Cp18S and Hs18S transcripts, respectively. Reactions were performed in hard-shell 384-well skirted PCR plates (Bio-Rad Laboratories, Hercules, CA) (10 μL/well) containing 3 μL diluted cell lysate, 5 μL one-step SYBR green master mix, 0.2 μl RT master mix and the following primers: Cp18S-1011F (5′-TTG TTC CTT ACT CCT TCA GCA C-3′) and Cp18S-1185R (5′-TCC TTC CTA TGT CTG GAC CTG-3′) primer pair for Cp18S rRNA (GenBank accession number: NC_006986.1), and Hs18S-1F (5′-GGC GCC CCC TCG ATG CTC TTA-3′) and Hs18S-1R (5′-CCC CCG GCC GTC CCT CTT A-3′) primer pair for Hs18S rRNA (GenBank accession number: NR_003286). Among them, Cp18S-1011F and Cp18S-1185R were described in our earlier studies reporting the original qRT-PCR assay and the efficacy of S-adenosylhomocysteine hydrolase inhibitors against the growth of *C. parvum in vitro* (Cai et al., 2005; Ctrnáctá et al., 2010), whereas Hs18S-1F and Hs18S-1R represented our newly optimized primers (that were also described in one of our more recent publications (Zhang et al., 2012). Hs18S levels were used as controls and for normalization. All reagents for the qRT-PCR were loaded using an epMotion 5070 automated pipetting system (Eppendorf, Hauppauge, NY).

Real-time qRT-PCR reactions were performed by a Bio-Rad CFX384 Touch Real-Time PCR Detection System. The reactions started with synthesizing cDNA at 50°C for 20 min, followed by 5 min at 95°C to denature RNA-cDNA hybrids and deactivate reverse transcriptase, and 40 two-temperature thermal cycles of PCR amplification at 95°C, 10 s and 58°C, 30 s. At the end of PCR amplification, melting curve analysis was performed between 65 and 95°C. At least two technical replicates were included in qRT-PCR reactions for each sample.

After qRT-PCR reactions were completed, amplification curves and melting peaks were examined to assess the quality and specificity of the reactions, followed by the computation of relative parasite loads based on the cycle threshold (C_T_) values of Cp18S and Hs18S transcripts as previously described. Briefly, the means of C_T_ values from technical replicates were first obtained for individual biological replicates, which were used to compute ΔC_T_ values between Cp18S and Hs18S (i.e., ΔC_T_ = C_T[Cp18S]_-C_T[Hs18S]_), and ΔΔC_T_ values between each experimental sample and control (i.e., ΔΔC_T_ = ΔC_T[sample]_-ΔC_T[control]_). Standard curves were generated by plotting ΔC_T_ values against the logarithm of the oocyst numbers used to inoculate cells, followed by linear regression to obtain the slope value (parameter A in Equation below) to calculate the detection efficiency and parasite loads using the following equation as described (Cai et al., 2005):

$$\begin{array}{l} Inhibition\left(\text{\%}\right)=\left(1-10^{A\cdot \Delta \Delta C_{T}}\right)\cdot \left(100\right) \end{array}$$

Because all samples contained much lower levels of Cp18S rRNA than those of Hs18S rRNA, we used two different dilution factors (i.e., 100- and 2000-fold dilutions of lysates for detecting Cp18S and Hs18S, respectively) as described above to maintain the linearity of detections. These dilution factors needed to be considered in calculating the relative level between Cp18S and Hs18S in a specified sample:

$$\begin{array}{l} Relative\left[Cp18S\right]=\frac{\left[Cp18S\right]}{\left[Hs18S\right]}=\left(\frac{100}{2000}\right)\cdot 10^{A\cdot \Delta C_{T\left[Cp18S-Hs18S\right]}} \end{array}$$

However, the effect of the dilution factors was eliminated in subsequent computation of the relative growth of *C. parvum* (Cp) between a sample (s) and control (c) based on ΔΔC values:

$$\begin{array}{l} Relative\;Cp\;growth=\frac{\left(\frac{\left[Cp\text{18}S\right]s}{\left[Hs\text{18}S\right]s}\right)}{\left(\frac{\left[Cp\text{18}S\right]c}{\left[Hs\text{18}S\right]c}\right)}=\frac{\left(\frac{100}{2000}\right)\cdot 10^{A\cdot △C_{T\left[s\right]}}}{\left(\frac{100}{2000}\right)\cdot 10^{A\cdot △C_{T\left[c\right]}}}\\ \;=10^{A\cdot △△C_{T\left[s\text{-}c\right]}} \end{array}$$

### Evaluation of the growth of host cells and parasite in 96-well plates

We also evaluated the growth of *C. parvum* in 96-well plates by qRT-PCR and microscopy, in which HCT-8 cells were cultured and infected with *C. parvum* as described above. For qRT-PCR analysis, total RNA was isolated from cells using an RNeasy RNA isolation kit (Qiagen), rather than directly using cell lysates, to ensure more precise measurements of the levels of Cp18S and Hs18S transcripts. RNA samples included those from cells infected with *C. parvum* for 3 and 44 h, respectively, and from uninfected cells cultured under the same condition and for the same time periods. For microscopic examination, sterilized glass coverslips were gently broken into pieces and placed into selected wells before seeding host cells. Cells infected with parasites for 3 and 44 h were fixed in 3.7% paraformaldehyde and stained with a rabbit polyclonal antibody against total *C. parvum* membrane proteins following standard protocols (Chen et al., 2003; Zeng et al., 2006). Samples were then mounted onto glass microscopic slides with a Anti-Fade mounting medium containing 4′,6-diamidino-2-phenylindole (DAPI) for counter-staining nuclei (Molecular Probes, Invitrogen), and examined under Olympus BX51 Research Microscope.

### Assay quality assessment

In addition to determining the linear dynamic range from standard curve derived from samples infected with varied numbers parasites, the uniformity and signal variability of the assay were also validated according to the “Assay Guidance Manual” (see chapter “HTS Assay Validation” at http://www.ncbi.nlm.nih.gov/books/NBK53196/) (Iversen et al., 2012). This method is recommended by the National Center for Advancing Translational Sciences (NCATS), National Institutes of Health (NIH) (see “Pre-Clinical Research Toolbox” at http://www.ncats.nih.gov/expertise/preclinical). In this study, inter-day tests were performed by three independent experiments. Each experiment used three 96-well plates to assess inter-plate variations, and each plate contained three experimental groups, which included uninfected HCT-8 cells (“Min” background signal), cells infected with parasites but untreated (“Max” signal), and cells that were infected with parasites and treated with 120 μg/mL (168 μM) of PRM (“Mid” signal). These groups (32 wells each) were arranged in an interleaved-signal format according to the Assay Guidance Manual.

The cultivation of host cells and *C. parvum in vitro*, preparation of cell lysates, and qRT-PCR assay were performed as described above. PRM was added into specified wells after the invasion and the removal of uninfected parasites. At the same time, DMSO at 0.5% final concentration was added into all wells. Based on the qRT-PCR data, mean (*AVG*), standard deviation (*SD*), and coefficient of variation (*CV*) were calculated for each group in each plate. Signal window (*SW*) and Z′ factor for each plate were calculated based on the Assay Guidance Manual as follows (Iversen et al., 2012):

$$\begin{array}{l} SW=\frac{\left({AVG}_{max}-3{SD}_{max}∕\sqrt{n}\right)-\left({AVG}_{min}+3{SD}_{min}∕\sqrt{n}\right)}{{SD}_{max}∕\sqrt{n}} \end{array}$$

$$\begin{array}{l} Z^{{}^{\prime }}=\frac{\left({AVG}_{max}-3{SD}_{max}∕\sqrt{n}\right)-\left({AVG}_{min}+3{SD}_{min}∕\sqrt{n}\right)}{{AVG}_{max}-{AVG}_{min}} \end{array}$$

### Small-scale drug screening

The assay is currently used in our laboratory to test drug efficacy and screen compound libraries against *C. parvum in vitro*. Here we report the data on 48 small molecules designated as C001–C048 to provide a snapshot on the assay performance. HCT-8 cells were cultured in 96-well plates and infected with *C. parvum* using procedures described above. In primary screening, drugs dissolved in DMSO were added into the culture immediately after the medium exchange at 3 h post-infection (hpi) time (10 μM final concentration). Each plate contained five negative controls without receiving drug treatment and three positive controls treated by 100 μg/mL (140 μM) of PRM. Each well, including negative and positive controls, contained 0.5% DMSO. At 44 hpi time, cell lysates were prepared using Bio-Rad iScript lysis buffer and the anti-cryptosporidial efficacies of drugs were evaluated by qRT-PCR as described above. Compounds that inhibited >65% of the parasite growth were re-tested. Two of the most effective drugs were then selected to generate dose-response curves to determine IC_50_ values.

## Results and discussions

### Growth of host cells and parasites in 96-well format in the 44-h infection assay

Similar to other apicomplexans, *Cryptosporidium* sporozoites invade host cells and undergo at least two generation of asexual cell cycles (merogony) before entering sexual development (gametogenesis and fertilization between macro- and micro-gametes) to form oocysts. We have observed that most *C. parvum* cultured in 24- or 48-well plates may complete the second generation of merogony and a large number of free merozoites may be released into the culture medium at 48 hpi. This complicates the quantification of parasite loads because free merozoites in the medium may be easily washed off during the sample preparation. To minimize this problem, our laboratory uses the 44 h infection assay, in which the majority of the intracellular parasites are in the late stage of meronts and only a minimal number of merozoites are present in the medium. To confirm that *C. parvum* cultured in 96-well plates followed similar invasion and intracellular developmental timeline, we compared the parasite growth at 3 and 44 hpi by qRT-PCR and fluorescence microscopy. We also evaluate the host cell growth at the time points correlated to the 3 and 44 hpi by qRT-PCR.

**Figure 1:**
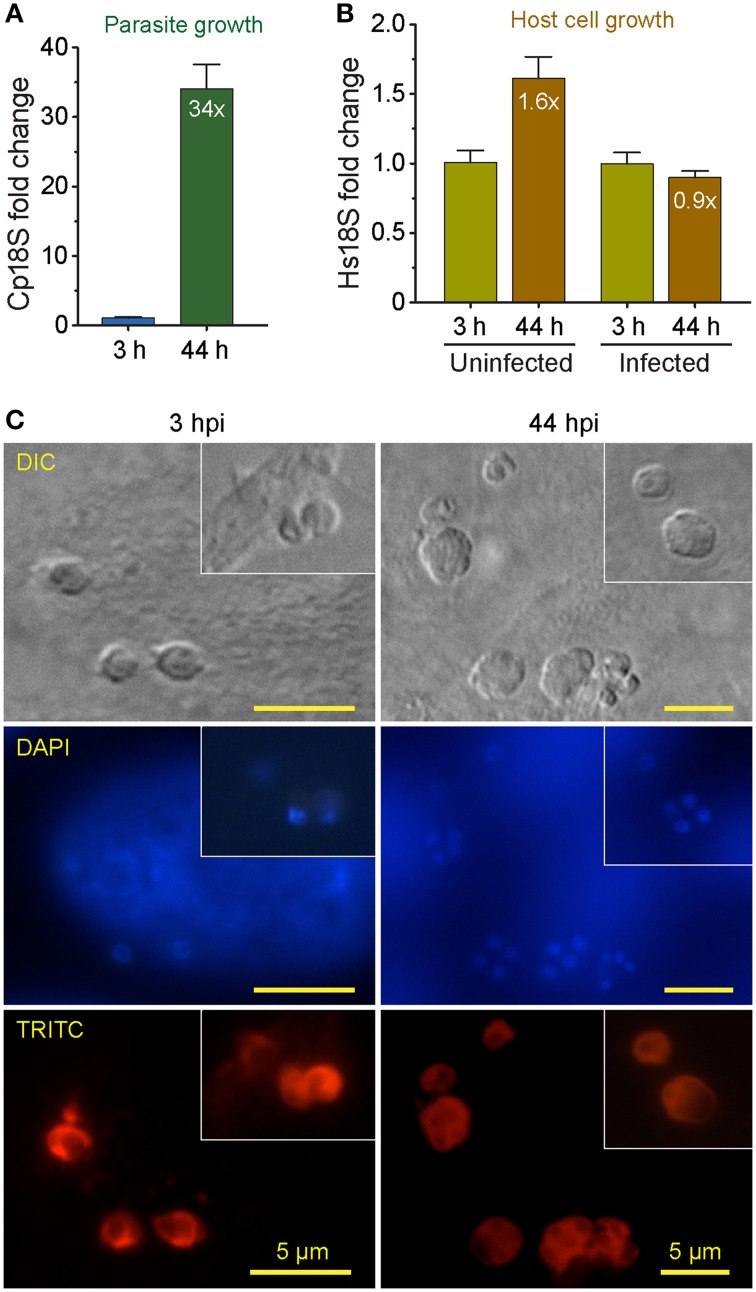
**Comparison of the growths between *Cryptosporidium parvum* and host cells (HCT-8 cell line) cultured in 96-well plates for 44 h post-infection (hpi) as determined by detecting their relative levels of 18S rRNA transcripts**. **(A)** The growth rate of *C. parvum* in HCT-8 cells between 3 and 44 hpi. The levels of 18S rRNA transcripts from the parasite (Cp18S) and host cells (Hs18S) were determined by qRT-PCR, and those of Hs18S were used for normalization before calculating the fold changes in Cp18S transcripts. **(B)** The relative levels of Hs18S in HCT-8 cells infected with *C. parvum* for 3 and 44 hpi. Uninfected cells were grown in parallel under the same condition for the same time periods. Bars represent standard error of the mean (SEM, *n* = 9) from three independent experiments. **(C)** Micrographs showing the development of *C. parvum* cultured in HCT-8 cells at 3 and 44 h post-infection (hpi) time points, in which intracellular parasites were labeled with a rabbit antiserum against *C. parvum* sporozoite total membrane proteins and a TRITC-conjugated goat anti-rabbit IgG secondary antibody and counter-stained with DAPI for nuclei. DIC, differential inference contrast microscopy. DAPI, 4′, 6-diamidino-2-phenylindole for counterstaining of nuclei. TRITC, Tetramethylrhodamine.

Using qRT-PCR, we have observed rapid intracellular parasite growth (i.e., 34-fold increase of Cp18S rRNA from 3 to 44 hpi) (Figure 1A), which gave us a good dynamic range to quantify the relative parasite growth. For comparison, there was only ~1.6-fold increase of uninfected Hs18S cultured for the same time period (Figure 1B), indicating that parasite grew >20 times faster than the HCT-8 cells in this cultivation system. We also observed that parasite infection slightly reduced the HCT-8 cell growth (i.e., ~10% decrease based on Hs18S rRNA levels) (Figure 1B), which might be attributed by the arrest of host cell growth upon infection and cell death upon the completion of merogony development and the release of parasite merozoites as reported by other investigators (Dobbelaere and Küenzi, 2004; Brunet et al., 2008).

DIC microscopy and IF labeling revealed the successful invasion of *C. parvum* sporozoites into the host cells after 3-h initial incubation of oocysts with host cells, in which the elongated sporozoites were transformed to small round trophozoites (Figure 1C, 3 hpi panel). However, it was possible that some sporozoites might have attached to, but failed to invade into host cells or undergo further development at the 3 hpi time point. The growth of parasite into late stage of meronts as evidenced by the presence of multiple nuclei in many meronts by DAPI staining (Figure 1C, 44 hpi panel). Intracellular parasites in these stages were also clearly visualized by IF staining using a rabbit antiserum against *C. parvum* sporozoite membrane proteins (Figure 1C, TRITC staining). These observations indicated that parasite cultured in 96-well plates exhibited an intracellular development timeline similar to those cultured in 24- and 48-well plates. Because majority of the parasites still remained intracellular at 44 hpi, they could be lysed together with host cell monolayers for RNA extraction. However, we still included a plate centrifugation step before washing cell monolayers and preparing cell lysates to ensure any free merozoites released into the medium were not excluded from subsequent analysis.

### Optimization the sample preparation used for simplified qRT-PCR

Several commercial lysis buffers are currently available for preparing cell lysates suitable for qRT-PCR without further purification of RNA (Ho et al., 2013). At the early of the project, we briefly evaluated Ambion Cells-to-cDNA™ II kit (Life Technologies) and iScript™ RT-qPCR Sample Preparation Reagent (Bio-Rad), and observed slight better performance in releasing intracellular parasite RNA by the Bio-Rad's reagent (data not shown) that was then used in our subsequent assay development. We also observed that standard sample preparation procedures recommended by the manufacturer was insufficient to release parasite RNA, largely because intracellular parasites were contained in parasitophorous vacuoles and protected by extra layers of membranes. We hence performed experiments to determine the optimal conditions including the amounts of lysis buffer and the addition of vortex and heating steps.

**Figure 2:**
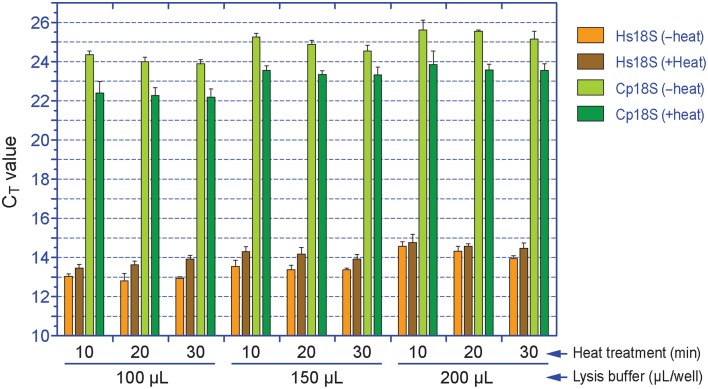
**Effects of the amount of lysis buffer and heat treatment on the release of RNA from *Cryptosporidium parvum* and HCT-8 cells**. Cell lysates were first diluted by 100 and 2000 times with nuclease-free water prior to qRT-PCR detection for Cp18S and Hs18S rRNA transcripts, respectively. The plotted C_T_ values were not calibrated to equal volume of lysis buffer. Bars represent standard error of the mean (SEM, *n* = 6). Heat treatment vs. un-treatment control, *p* < 0.005 by Student's *t*-test in all samples.

We observed that heat treatment of cell lysates at 75°C for 10–30 min significantly improved the release of parasite RNA. When samples were lysed in l00, 150, or 200 μL/well lysis buffer, heat treatment for 10, 20, or 30 min reduced the C_T[Cp18S]_ values from 24.4, 24.0, and 23.9 to 22.4, 22.3, and 22.2 in the 100 μL/well group, from 25.3, 24.9, and 24.5 to 23.6, 23.4, and 23.3 in the 150 μL/well group, or from 25.6, 25.5, and 25.2 to 23.9, 23.6, and 23.6 in the 200 μL/well group, respectively (*p* < 0.005 by Student *t*-test in all samples in comparison to the unheated counterparts) (Figure 2). However, we also observed certain levels of heat-induced host cell RNA degradation (i.e., C_T[Hs18S]_ values in heated samples were increased by ~0.2–1.0) (Figure 2). One possible explanation was that the host cell membranes could be rapidly lysed to release RNA with ice-cold lysis buffer (as heating was not included in the manufacturer's protocol), and there was a small window of time at the beginning of the heating process for the host cell RNases to become active before being deactivated by heat. On the other hand, more parasite RNA was released after the temperature reached to 75°C and RNases were deactivated. However, the final host cell RNA concentrations appeared to be consistent within each experimental group (i.e., CV values at 0.75–3.04% for all groups), indicating that the RNA degradation would not result in inconsistency in subsequent determination of relative Hs18S levels. Finally, based on the observation that heating between 10 and 20 min in wells containing 200 μL lysis buffer resulted in the least degradation of host cell RNA (ΔC_T_ at 0.20–0.25), but the most improvement in releasing parasite RNA (ΔC_T_ at −1.8 to −2.0), we determined that the optimal condition was the use of 200 μL iScript™ RT-qPCR Sample Preparation Reagent and heating for 15 min.

**Figure 3:**
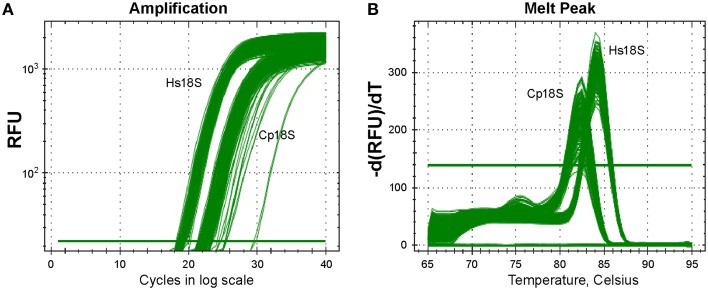
**Specific detection of Cp18S and Hs18S rRNA transcripts as demonstrated by amplification curve (A) and melting curve (B) analyses of a representative plate**. Lysates of HCT-8 cells grown in 96-well plates and infected with *Cryptosporidium parvum* for 44 h were used in qRT-PCR reactions in 384-well format.

The subsequent qRT-PCR amplification protocol in 384-well format using cell lysates was effective and specific based on agarose gel analysis of amplicons (data not shown) and post-run analysis of amplification and melting curves (Figure 3). In the melting curves, Cp18S and Hs18S displayed two strong and distinguishable peaks, although there was a small minor peak in the Cp18S curves (Figure 3B).

### Assay validation on plate uniformity, linear dynamic range, and drug testing

Three experimental cell culture groups were used in assay validation (i.e., a total of 864 uninfected, infected/untreated, and infected/PRM-treated samples in nine plates). For uninfected cell samples containing that should not contain any Cp18S transcripts, the determined Cp18S C_T_ values were resulted from low level non-specific amplification due to the lack of specific templates and/or self-amplification of primers, which was similar to the background signals produced in negative controls without enzyme in a biochemical assay. Under the conditions used in the assay, the overall mean of C_T_ values for Cp18S from uninfected cells in all plates was 30.14 (between 29.34 and 31.07). These C_T_ values were much higher than those derived from cells infected with *C. parvum* (mean = 22.15, between 21.40 and 22.90) (Table 1). Because of the negative correlation (inversely proportional) relationship between C_T_ values and template concentrations, the higher C_T_ values in uninfected cells indicated that the noise signals in this assay (i.e., background signals in negative controls in the absence of parasites) were roughly >250-fold lower than the signals from the infected cells (i.e., noise levels equivalent to 0.4% of the signals). For Hs18S transcripts, the overall mean of C_T_ values from all groups was 13.46 (between 12.92 and 14.06). In the infected/untreated groups, the average ΔC_T_ value difference between those of Hs18S and Cp18S was 8.6 (Table 1). After considering the dilution factors (i.e., cell lysates used for detecting Hs18S and Cp18S transcripts were diluted by 100 and 2000 folds, respectively), these C_T_ values suggested the presence of 7000–8000-fold more Hs18S than Cp18S in a typical sample.

**Table 1:** **Summary of the C_T_ values in the uniformity assay using the simplified qRT-PCR assay^a^**.

**Exp**	**Plate**	**Infected, untreated**				**Infected, PRM-treated**				**Uninfected, untreated**			
		**Cp18S**		**Hs18S**		**Cp18S**		**Hs18S**		**Cp18S**		**Hs18S**	
		**Mean**	**SD**	**Mean**	**SD**	**Mean**	**SD**	**Mean**	**SD**	**Mean**	**SD**	**Mean**	**SD**
1	1	22.24	0.17	13.93	0.17	23.32	4.31	13.55	2.55	30.70	0.25	13.58	0.31
1	2	22.57	0.20	13.77	0.19	23.85	0.30	13.88	0.18	31.07	0.26	12.92	0.30
1	3	22.90	0.53	14.06	0.23	24.47	0.41	13.88	0.25	30.68	0.19	13.69	0.19
2	1	21.67	0.25	13.49	0.25	23.29	0.33	13.51	0.22	30.15	0.29	13.51	0.20
2	2	21.40	0.25	13.32	0.18	23.17	0.27	13.25	0.22	29.68	0.27	13.36	0.29
2	3	21.94	0.52	13.45	0.34	23.65	0.43	13.42	0.34	30.08	0.26	13.42	0.36
3	1	21.93	0.36	13.02	0.38	23.59	0.31	12.99	0.28	29.78	0.26	13.09	0.38
3	2	22.25	0.27	13.33	0.29	23.83	0.31	13.27	0.27	29.34	0.24	13.30	0.25
3	3	22.49	0.41	13.57	0.34	24.09	0.26	13.45	0.30	29.79	0.40	13.44	0.28

a *All mean values are C_T_ values derived from each plate in three independent cell cultivation experiments (Exp) performed in different dates. PRM, paromomycin at 120 μg/mL (168 μM)*.

Using the validation protocols recommended by NIH/NCATS, we observed high intra-plate, inter-plate, and inter-day uniformity for the assay (Table 2). All CV values were < 5%, much better than the 20% threshold recommended by the Assay Guidance Manual. No significant inter-plate and inter-day data shifts were observed. The SW and Z′ values in the nine plates were ranging from 17.35 to 44.14 and 0.73 to 0.87, respectively. Both values were much higher than the recommended acceptance criterions (i.e., SW ≥ 2 and Z′ ≥ 0.4). The excellence of the assay is largely due to high specificity in the RT-PCR amplification, resulting in a ~256-fold signal difference between infected and uninfected samples as described above. In the PRM-treated groups, the classic anti-cryptosporidial drug inhibited the parasite growth by 66.07–71.46% in all plates except for the plate 2 in experiment 1 that was at a slightly lower level of 54.90% (Table 2). These values were consistent with our *in vitro* drug efficacy tests using total RNA samples isolated by traditional column-based isolation kits from cells cultured in 24- and 48-well plates (Cai et al., 2005; Zhang et al., 2012; Yu et al., 2014).

**Table 2:** **Assay validation on the uniformity of the simplified qRT-PCR assay based on normalized ΔC_T_ values^a^**.

**Exp**	**Plate**	**Infected, untreated**			**Infected, PRM-treated**			**Uninfected, untreated**			**SW**	**Z′**	**Efficacy of PRM**	
		**Mean**	**SD**	**CV (%)**	**Mean**	**SD**	**CV (%)**	**Mean**	**SD**	**CV (%)**			**% Inh**	**SD**
1	1	8.31	0.23	2.00	10.08	0.40	2.00	17.12	0.35	1.45	30.40	0.86	69.54	7.84
1	2	8.79	0.20	1.61	9.97	0.32	1.61	18.15	0.36	1.42	31.74	0.87	54.90	10.49
1	3	8.84	0.49	3.94	10.60	0.39	3.94	16.99	0.21	0.89	44.14	0.82	69.34	7.81
2	1	8.18	0.36	3.13	9.78	0.36	3.13	16.64	0.40	1.72	23.94	0.81	66.07	8.30
2	2	8.08	0.36	3.16	9.93	0.33	3.16	16.32	0.38	1.65	24.80	0.81	71.46	6.35
2	3	8.49	0.47	3.95	10.24	0.64	3.95	16.67	0.48	2.02	18.26	0.75	67.30	13.58
3	1	8.91	0.53	4.24	10.59	0.46	4.24	16.68	0.45	1.89	18.06	0.73	67.20	11.32
3	2	8.92	0.31	2.47	10.57	0.44	2.47	16.04	0.33	1.45	24.79	0.81	66.67	10.19
3	3	8.92	0.47	3.77	10.65	0.39	3.77	16.37	0.45	1.94	17.35	0.74	68.82	8.70

a *All mean values are* Δ*C_T_ values between Cp18S and Hs18S derived from each plate in three independent cell cultivation experiments (Exp) performed in different dates. % Inh, % inhibition; PRM, paromomycin at 120 μg/mL (168 μM); SW, signal window; Z′, Z′ score*.

**Figure 4:**
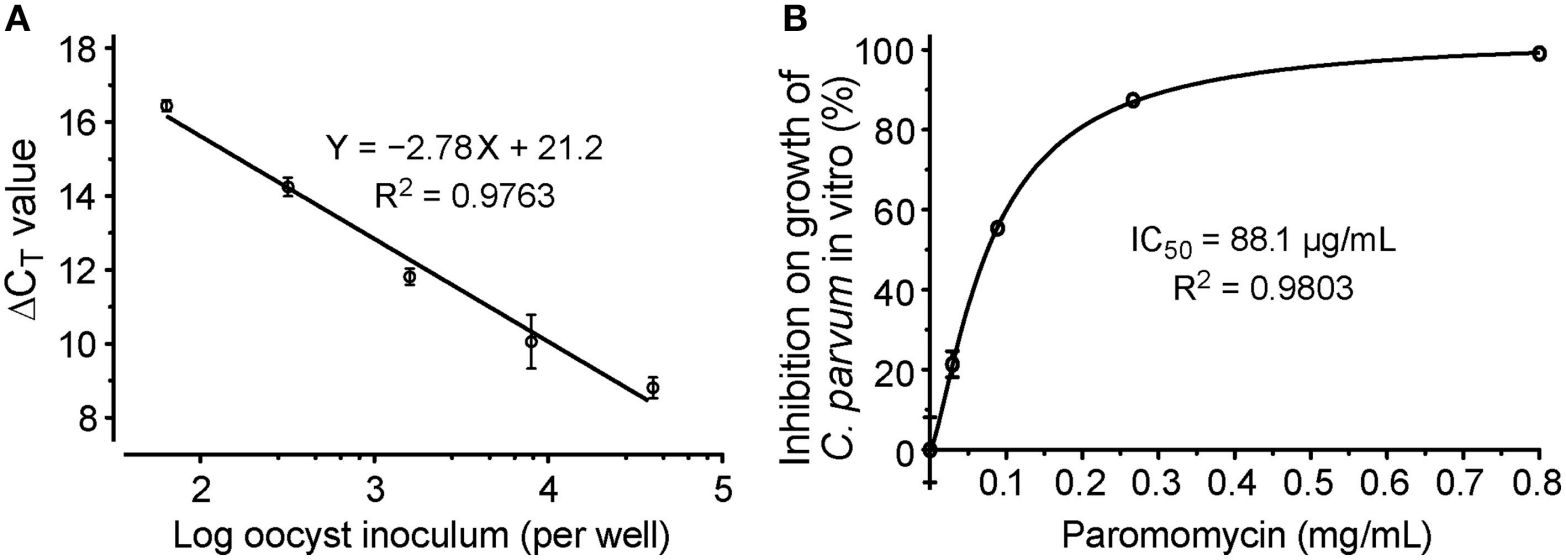
**Evaluation of drug efficacy using the new qRT-PCR assay**. **(A)** Standard curves showing the relationship between the number of inoculated oocysts and ΔC_T(Cp18S−Hs18S)_ values; **(B)** Dose-response curve on the *in vitro* anti-cryptosporidial activity of paromomycin. Bars represent standard errors of the mean (SEM) derived from at least two biological replicates.

We also generated standard curves from cells infected with various numbers of parasites. We considered that this approach was better than the traditional method using serially diluted RNA templates, because standard curves derived from various numbers of infected parasites would correct systematic errors from infection, sample preparation to qRT-PCR procedures, rather than to only correct the errors introduced at the qRT-PCR step. In this assay, linear relationship was observed between the ΔC_T_ values and the samples isolated from cells infected with 64–4 × 10^4^ oocysts per well (Figure 4A). The ΔC_T_ values from samples infected with more or less parasites frequently fell outside of the linear range (data not shown). The linear dynamic range for this new assay (~156-fold) was smaller than our previously reported assay using conventional protocols (Cai et al., 2005). There were two possible explanations. Firstly, the 96-well format had lower limits on the number of parasites that could be cultured in 96-well without density effect. Secondly, in comparison to conventional RNA isolation methods, cell lysates contained much less RNA due to the less efficiency in releasing RNA by the lysis buffer and higher dilutions needed to minimize the inhibition of RT-PCR reactions caused by substances present in the lysis buffer. However, the 156-fold linear dynamic range was sufficient for testing drug efficacy *in vitro*, as it could theoretically resolve < 1% changes of parasite loads in the assay system.

We further evaluated the assay by testing the dose-response of PRM on the parasite growth, in which the drug at concentrations from 30 to 800 μg/mL reduced the growth of *C. parvum* by 19–97%. Using a non-linear curve fit with a sigmoidal model, the IC_50_ value was determined at 88.1 μg/mL (123 μM) (Figure 4B). These inhibition values were comparable to those obtained in our previous report at 89.7 μg/ml (Cai et al., 2005), suggesting that the assay was suitable for determining the inhibitory kinetics and IC_50_ values of anti-cryptosporidial compounds identified by HTS.

### Testing the assay by a small scale drug screening

**Figure 5:**
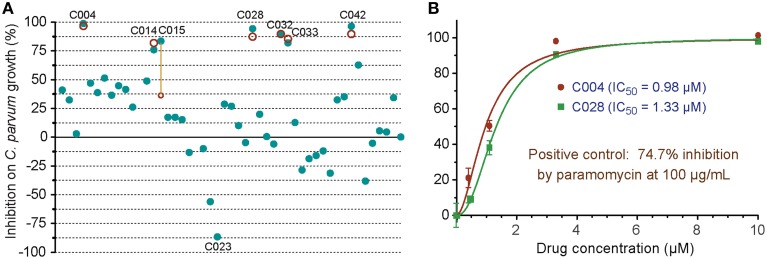
***In vitro* anti-cryptosporidial activities of 48 small molecules (C001–C048)**. **(A)** Efficacies of all 48 compounds (10 μM each) obtained from primary screening (solid teal round dots). A second test was performed on seven top hits (red circles), in which six compounds displayed similar levels of efficacy between the two tests as shown by overlaps or near distances of their corresponding teal dots and red circles. Only one compound showed significantly different efficacies between the two tests (highlighted by a yellow line to link the corresponding teal dot and red circle). Compound codes are given on the seven top hits and one of the potential parasite growth enhancer as discussed in the text. **(B)** Dose-response curves on the anti-cryptosporidial activities of C004 and C028 compounds. Paromomycin at 100 μg/ml (140 μM) was used as a positive control in this assay. Bars represent standard errors of the mean (SEM) derived from at least two biological replicates.

In the primary screening of 48 small molecules (10 μM each), two compounds (C012 and C020) displayed cytotoxicity on HCT-8 cells, while others displayed a wide range of effects on the *C. parvum* growth *in vitro* (i.e., from −86.6 to 98.8% inhibition) (Figure 5). The negative inhibition values suggested that a few compounds might actually promote the parasite growth and/or affected more on host cells than on parasites, which could be evaluated by looking at the Hs18S and Cp18S C_T_ values. For example, for C023 with −86.6% inhibition, the mean C_T_ values for Hs18S and Cp18S were 13.38 and 18.08 (vs. 13.63 and 19.17 in the DMSO control), suggesting that this compound likely promoted the parasite growth more than reducing the host cell growth. Because we were interested only in identifying anti-cryptosporidial inhibitors in this study, we repeated the primary testing on the seven most efficacious compounds, in which six compounds exhibited similar efficacies (Figure 5A, red circles). We also performed dose-response experiments on C004 and C028, in which both compounds at 10 μM displayed the same efficacy as observed in the two primary testing experiments (Figure 5B). Their IC_50_ values were at 0.98 and 1.33 μM, respectively, suggesting that these two compounds might be further explored for drug development or serve as leads. Experiments to further characterize these two compounds and other top hits from the 48 compounds are still ongoing as part of our larger effort in discovering new anti-cryptosporidial drugs, which will be reported separately. Nonetheless, these observations conformed the robustness and suitability of the assay in high-throughput screening of drugs by directly evaluating their anti-cryptosporidial efficacy *in vitro*.

## Conclusions

We have developed a simplified real-time qRT-PCR assay suitable for high-throughput or high-volume screening of drugs against the growth of *C. parvum in vitro*. This was achieved by directly using cell lysates in qRT-PCR amplification. The assay was adapted and optimized in 96-well format for culturing parasite and 384-well format for SYBR-green based one-step real-time RT-PCR amplification and detection. Intra-plate, inter-plate, and inter-day uniformities were validated by NIH/NCATS recommended tests (i.e., SW and Z′ values = 17.35–44.14 and 0.73–0.87, respectively). The assay exhibited >150-fold linear dynamic range using samples isolated from cells infected with serially diluted parasites. The robustness and effectiveness of the assay were also confirmed by evaluating the anti-cryptosporidial efficacy of paromomycin, and by a small scale screening of 48 compounds.

### Conflict of interest statement

The authors declare that the research was conducted in the absence of any commercial or financial relationships that could be construed as a potential conflict of interest.
